# Determinants of Obesity and Metabolic Health in the Afghan Population: Protocol, Methodology, and Preliminary Results

**DOI:** 10.1007/s44197-021-00026-0

**Published:** 2022-01-07

**Authors:** Mohammad Sediq Sahrai, Inge Huybrechts, Carine Biessy, Sabina Rinaldi, Pietro Ferrari, Abdul Wahed Wasiq, Marc J. Gunter, Laure Dossus

**Affiliations:** 1 Nutrition and Metabolism Section, International Agency for Research On Cancer (IARC/WHO), 150 Cours Albert Thomas, 69372, Cedex 08 Lyon, France; 2 Department of Internal Medicine, Faculty of Medicine, Kandahar University, 3801 Kandahar, Afghanistan

**Keywords:** Dietary patterns, Obesity, Metabolic health, Afghanistan

## Abstract

**Background:**

Non-communicable diseases (NCDs) cause more than 70% of deaths worldwide and share modifiable risk factors including obesity and metabolic abnormalities. Over the past 15 years, many changes in lifestyle, dietary patterns, physical activity, and socioeconomic status have been observed in the Afghan population. This study aims to investigate which specific lifestyle factors, dietary patterns, and characteristics of Westernization are associated with an increased risk of being overweight or obese and with poor metabolic health in the Afghan population.

**Methods:**

A population-based cross-sectional study was conducted where a total of 729 male and female participants were recruited. Face-to-face interviews and anthropometric measurements were conducted by trained health staff using standardized questionnaires which included information on socio-demographic and housing characteristics, income, occupation, ethnicity, personal and family medical history, stress, anthropometry, diet, and physical activity. Bioelectric impedance analysis (BIA) was used to estimate body composition, including overall body fatness. Physical activity was measured using the short version of the International Physical Activity Questionnaire (IPAQ). For a comprehensive assessment of dietary intake, a food-frequency questionnaire (FFQ) specific to the Afghan population was developed which included all local food items relevant to the population. Lipid profile and fasting glucose were measured in a local laboratory. Biospecimens were collected using dried blood spots (DBS) and dried stool cards to perform microbiome and biomarker-based research.

**Discussion:**

This is the first study which will assess dietary patterns, lifestyle factors, and their association with obesity and metabolic health in Afghanistan. Such a study will aid the development of dietary and lifestyle guidelines in Afghanistan which will promote better health and educate people to make healthy food choices. The findings will also help in designing and implementing effective public health strategies to promote a healthy lifestyle and prevent the epidemic of overweight and obesity, and, hence, reduce the burden of non-communicable diseases in the region.

## Introduction

Non-communicable diseases (NCDs) are responsible for more than 70% of deaths worldwide and have shared modifiable risk factors such as unhealthy diet, physical inactivity, tobacco, and alcohol use. Some of these risk factors lead to obesity and metabolic health abnormalities [1]. Obesity is a major public health problem worldwide and its prevalence has almost tripled over the last 45 years [2]. Major determinants of obesity are high energy intakes (in particular high energy dense foods) and low energy expenditures [3]. In Low- and Middle-Income countries (LMICs) experiencing nutrition transition, including Afghanistan, there is often the co-existence of undernutrition and overnutrition across the life course which may lead to specific food and health patterns [4].

Over the last 15 years, many changes have been observed in the lifestyle, dietary patterns, physical activity, and socioeconomic status of the Afghan population. However, little is known on the specific determinants for obesity and poor metabolic health in Afghanistan. Due to rapid economic, social, and cultural changes in Afghanistan, dietary patterns are shifting from the traditional diets and lifestyles to more westernized habits with a marked increase in consumption of energy rich foods. As a consequence, the incidence of non-communicable diseases such as diabetes, cardiovascular disease, and certain cancers is rising in Afghanistan where the burden of communicable diseases is already affecting the country [5]. However, there is currently no published study on dietary patterns characterizing the Afghan population and their association with obesity and metabolic health.

To collect valuable data on the diet and lifestyle habits of the Afghan population and their association with obesity and metabolic parameters, we have conducted a population-based cross-sectional study in Kandahar city, called Kandahar Obesity Research (KOR). We hypothesize that specific lifestyle factors and dietary patterns, and characteristics of Westernization are associated with an increased risk of being overweight or obese and with poor metabolic health. To address this hypothesis, the overall objectives of the study are

1. To develop a cross-sectional study focused on overweight, obesity, and metabolic health,
2. To identify determinants of obesity in this population,
3. To inform public health strategies to promote a healthy lifestyle and prevent the epidemic of overweight and obesity, and, hence, reduce the burden of non-communicable diseases in the region.

To do so, we

1. collected information on socio-demographic and housing characteristics, income, occupation, ethnicity, personal and family medical history, stress, anthropometry, and physical activity;
2. collected biological specimens, such as blood, urine, and stool;
3. will evaluate the associations of dietary patterns, physical activity, and other lifestyle factors with various anthropometric measurements;
4. will evaluate the associations of nutritional and metabolic biomarkers with various anthropometric measurements,
5. will investigate the role of microbiome on obesity.

To our knowledge, this is the first and largest population-based study conducted in Afghanistan on diet using a food-frequency questionnaire and with the collection of biological specimens for future biomarker-based research.

## Materials and Methods

### Study Setting and Design

Kandahar, one of the oldest cities in the world (founded by Alexander the Great in 330 BC), is located in the south of Afghanistan. The province has played a significant role in the history of Afghanistan due to its strategic location on the trade routes of central Asia. It is a major transit route to the Southern and Western Afghanistan, and to Indo-Pakistan and Persian Gulf outside of Afghanistan. Beside its commercial important, Kandahar also has a long history of developed and successful agriculture with one of the largest amount of agricultural land in Afghanistan [6]. Kandahar is the second largest province of Afghanistan after Kabul and is divided into 18 administrative units. The province has an estimated population of 1.4 million inhabitants of whom 0.52 million are living in urban areas [7].

A population-based cross-sectional study was conducted in the medical research unit of Kandahar University in Kandahar, Afghanistan. The entire study recruitment was carried out in 7 months from November, 2018 to May, 2019. The recruitment process for the survey was announced to the public by distributing study brochures as advertisements to the staff in Kandahar University, public health offices, Kandahar city municipality offices, and to all study participants at the end of their interviews. These brochures included all the information about the study in the local language and instructions for registration. The eligible criteria were: (1) male or female residents of Kandahar province (living for more than 1 year), (2) aged between 20 and 75 years, and (3) able to sign informed consent and provide biospecimens. Inability to provide informed consent, pregnant women, people with severe health diseases, and visitors from other provinces were excluded from the study. We use convenient sampling to recruit equal number of normal weight, overweight, and obese participants. At the end of the 7-month study period, a total of 729 participants were recruited from the population residing in the province, and after excluding underweight participants and those with missing nutritional assessment data, 711 were finally included in the study for statistical analyses.

### Sample Size

We will have statistical power greater than 80% at a significance level (a) of 5% to detect a correlation of 0.45 or higher between dietary intakes or biomarker levels and obesity based on 178 subjects, after accounting for stratification by gender and age and for multiple comparisons, assuming that *p* = 10 statistical tests will be performed. Lower correlation values will be detected in overall analyses or in analyses stratified by either age or sex. These values were computed the ‘Power’ procedure in SAS 9.4.

### Ethics

An informed consent form was developed in the Pashto language which included the information sheet and the consent certificate. This consent form, study protocol, and all the questionnaires were submitted to the IARC Ethics Committee, Kandahar University’s institutional review board (IRB) and FHI 360’s IRB. A consent form was obtained from each participant. A project description leaflet was developed in Pashto language and given to all participants before their meeting with trained staff, which clearly explained the scope of the study, the extent of participation, and the expected benefits of the study as a whole for better prevention of obesity.

### Measures and Data Collection

**Fig. 1:**
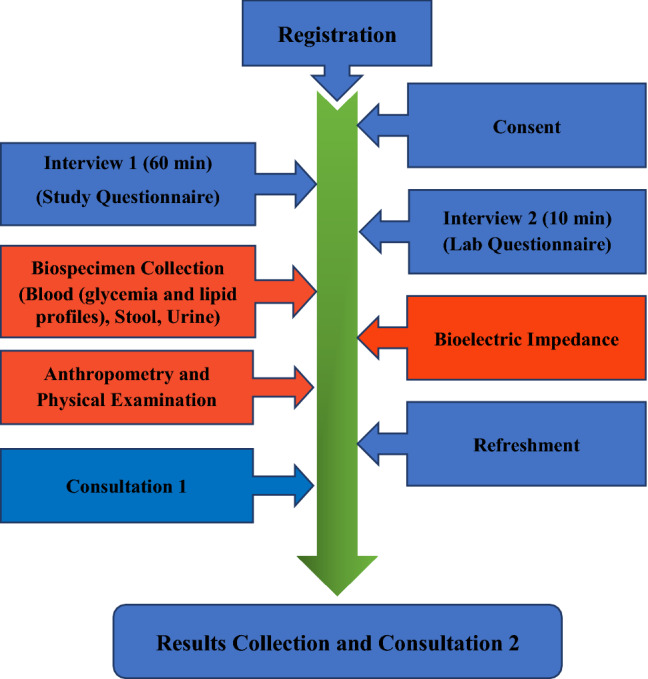
Flowchart of all study procedures: all registered participants (after signing the consent form) were interviewed (Interview 1) by a doctor using the study questionnaire. This main interview was followed by a second short interview (Interview II) with a questionnaire related to biospecimen collection by a laboratory specialist. All participants provided blood (fasting), stool and urine (10%) samples. Data regarding anthropometry, blood pressure measurement, abdominal ultrasound, and bioelectric impedance were also collected in a fasting state from all participants, and then, the participants were provided with refreshments. If the participants had any question regarding their health, these were discussed during consultation 1. All study participants received their results regarding blood glucose and lipid profile, H pylori in stool, liver scan (fatty liver disease), blood pressure, and BMI after 3 days of interview (Results Collection and Consultation 2)

Figure 1 describes the flowchart of all study procedures.

All the participants were provided with information about the study and were asked to provide written informed consent (in Pashto language). Face-to-face interviews and anthropometric measurements were conducted by trained health staff using standardized questionnaires. Training of health staff for interviews and study procedures was conducted in the medical faculty. Interviews and all the study procedures of the participants were undertaken in the medical research unit following Standard Operating Procedures (SOPs) specifically developed or adapted for the study. Table 1 summarises all the measurements of data collection in the study.

**Table 1:** Summary of measurements of data collection in KOR study

Examination	Measurements	Details
Face-to-face interview	Demographics and socioeconomic information	Sex, age, ethnicity, year of birth, address, housing characteristics, education, marital status, occupation, household income
	Lifestyle	Smoking, tobacco snuff, alcohol, physical activity
	Medical history	Cardiovascular, respiratory, digestive and urogenital diseases, cancer, diabetes, allergy, and oral health
	Family history of disease	High blood pressure, diabetes, cancer and others
	Reproductive history (women)	Age at menarche, age at menopause, contraception, reproductive organs surgery, parity, and breastfeeding
	Stress	Anxiety and depression symptoms (Hopkins Symptoms Checklist)
	Dietary groups	Dairy; meat, eggs and fish; fruits; vegetables; legumes; whole and refined cereals; nuts, seeds and dried fruits; fats and oil; fast food and snacks; sweets and desserts; beverages; spices and condiments
Physical Examination	Anthropometric measures	Weight, height, sitting height, body mass index (BMI), waist and hip circumferences, blood pressure, imaging (liver ultrasound), bioelectric impedance analysis (BIA)
Clinical lab-based tests	Blood	Total cholesterol (TC), triglycerides (TG), high- and low-density cholesterol lipoprotein (HDL-C, LDL-C), and fasting blood glucose
Bio-sample collection	Blood, stool, and urine	GenSaver, GenCollect, and BioSample TFN collection cards (Ahlstrom) to be stored at IARC for future biomarker analyses (i.e., metabolomics, inflammation markers, fatty acids, microbiome)

#### Non-dietary Questionnaire and Anthropometry

A standardized questionnaire was developed from other studies conducted in collaboration with IARC [8, 9] and administered by trained health workers in the research centre. It included information on socio-demographic and housing characteristics, income, occupation, ethnicity, personal and family medical history, stress, anthropometry, and physical activity. Socio-economic and demographic indicators include age, marital status, religion, number of siblings and children, education level, occupation, ethnicity (Pashtun, Tajik, Hazara, Uzbek, Baloch, others), residential address, type of housing, ownership of house, and other house items. Personal and medical history include smoking status, diseases during childhood, vaccination history, previous diseases, present chronic diseases, trauma, anxiety and depression [using Hopkins Symptoms Checklist (HSCL)], previous hospitalization, recent treatment for a disease, regular intake of any medicine or dietary supplements, use of herbs and medicine plants, oral health, family history of cancer and other chronic disease in first degree relative, and for women, parity, age at first pregnancy, duration of breastfeeding, menstrual cycle history (age at menarche, menopausal status), and use of oral contraceptive and hormone therapy.

Anthropometric measurements were performed by trained health personnel. Body weight was measured in all participants dressed in thin clothes with a digital electronic scale to the nearest 0.1 kg. Both standing and sitting heights were measured without shoes with a stadiometer to the nearest millimetre. Body mass index (BMI) was calculated as weight (kilograms) divided by standing height (meters) squared. We used WHO’s classification for BMI categories: normal weight (BMI of 18.5–24.9 kg/m^2^), overweight (BMI of 25.0–29.9 kg/m^2^), and obese (BMI of ≥ 30 kg/m^2^). Waist circumference was measured midway between the lowest rib and superior border of the iliac crest at the end of normal expiration using a non-elastic tape to the nearest millimetre. Hip circumference was measured in standing position at the level of the most prominent part of the gluteus. Waist/hip ratio (WHR) was calculated from these measurements. For abdominal obesity, a cut-off point of 80 cm and 94 cm and for waist/hip ratio a cut-off point of 0.85 and 0.90 were considered for women and men, respectively.

Bioelectric impedance analysis (BIA) was used (Nutriguard Data Input device) to estimate body composition, particularly body fat. All the BIA measurements were performed, while the person was lying in the supine position. To determine body fat (in kg and percent), lean mass (fat-free mass), body water, plus body cell mass (BCM) and extra-cellular mass (ECM), four electrodes were placed on the right hand, wrist, foot, and ankle, and were connected to a generator applying an alternating electrical current of 0.8 mA and 50 kHz.

Participants were also asked about changes in their body weight during the last year, weight control methods, and body silhouette at various ages. Pictograms with 9 options (from very thin to very fat), which are validated in different settings [10], were shown to both male and female participants to identify their body silhouette at different ages: Men, around ages 5–10 years, 15, 20, 30, 40, 50 years and at current age; Women, around ages 5–10 years, at menarche, 15, 20, before first birth, 30, 40, 50 years, at menopause and at current age.

Physical activity (PA) was measured using the short version of International Physical Activity Questionnaire (IPAQ) [11]. The questionnaire provided information about the duration of the physical activity in hours and minutes during a usual week. The intensity of physical activity was recorded as light, moderate, and vigorous physical activities and later converted into METS (metabolic equivalent).

#### Dietary Assessment

For a comprehensive assessment of dietary intake, a detailed country-specific food-frequency questionnaire (FFQ) was developed which included all local food items relevant to the population. Data from previous dietary monitoring surveys have been used to select the food groups of interest [5]. In addition, a research team of nutritional epidemiologists from IARC (International Agency for Research on Cancer) and Afghanistan checked the food list derived from these existing resources to optimize the food list and examples to be included further considering usual foods commonly consumed in Afghanistan. The food list of the final questionnaire consisted of different food items/categories (combining foods with similar food composition) and different consumption frequency categories (number of times per day, per week, per month, rarely or never). All foods consumed by the population in large and small quantity were included in the questionnaire. Different kinds of traditional and industrial beverages consumed by the population were also included in the questionnaire.

Each food item in the FFQ was assigned a portion size using standard local household units such as a plate, bowl, spoons of different sizes (tablespoon, teaspoon), tea-pot, tea-glass, and glass of water, as well as using photographs of foods and typical preparations of the local population, included in a food portion photograph book (FPPB) developed by the research team.

Regarding seasonal foods, participants were asked to answer the question based on intakes during periods/seasons when these foods were available. The daily intake was calculated according to the number of months per year that each seasonal food item was available.

The research team will also compile Food Composition Tables. For the compilation of the food composition tables, we will use the indirect method based on pre-existing data from literature and local/neighbouring regions. Our data will be compiled according to international standards and guidelines for food composition data set by the FAO/INFOODS [FAO/INFOODS (2012). FAO/INFOODS Guidelines for Checking Food Composition Data prior to the Publication of a User Table/Database-Version 1.0. FAO, Rome] [12]. The food composition table will be used to assess nutrient intakes from the FFQ.

The dietary intake assessment was performed by trained health staff who asked the participants to report their habitual consumption frequency of the different foods listed in the FFQ during the last year. The food portion photograph book showed life-size colour photographs of foods in four portion sizes with photographs of utensils. The frequency categories included in the questionnaire were: Never, < 1 time per month, 1–3 times a month, once/week, 2–4 times/week, 5–6 times/week, once/day, 2–3 times/day, 4–5 times/day, and 6 times/day.

Daily food intakes were calculated by multiplying the frequency of consumption with the selected portion size, considering also the number of months per year that seasonal foods were available.

#### Clinical Non-invasive Assessment

As no incentive was paid to the participants, the following non-invasive clinical measures were used to facilitate recruitment and as a compensation for the participant’s time and contribution. Blood pressure was measured by a mercury sphygmomanometer at right hand supported at heart level after sitting quietly for 15 min and recorded to the nearest 2 mmHg. Hypertension was defined as systolic blood pressure values of ≥ 140 mmHg and/or diastolic blood pressure values of ≥ 90 mmHg [13] or based on use of antihypertensive medicine. Abdominal ultrasound was performed by a trained physician. The purpose of the procedure was to scan the abdomen for any pathological changes. The liver was specifically scanned for the diagnosis of non-alcoholic fatty liver disease.

Participants were also evaluated for symptoms of anxiety and depression using Hopkins Symptoms Checklist (HSCL-25). This questionnaire contained 25 questions, where 10 and 15 questions were asked to assess anxiety and depression symptoms, respectively [14]. A 4-point Likert scale was used to score each symptom and the total score was divided by 25 (total number of symptoms). Participants with a mean score of ≥ 1.55 were considered probable psychiatric cases and those with a mean score of more or equal to standard cut-off of 1.75 were considered symptomatic [15].

The results of the study were shared with the participants and on their request, they were also provided with feedback about their diet, physical activity level, and blood lipid and glucose profile. In case of any abnormal findings, the participants were examined free of cost and treated similarly to other patients by a specialist according to the facilities present in the teaching hospital.

### Biological Specimen Collection

IARC’s standardized protocol for specimen collection was implemented as described in “Common Minimum Technical Standards and Protocols for Biobanks Dedicated to Cancer Research, IARC Technical Publication No. 44, 2017″ (http://publications.iarc.fr/Book-And-Report-Series/Iarc-Technical-Publications/Common-Minimum-Technical-Standards-And-Protocols-For-Biobanks-Dedicated-To-Cancer-Research-2017)”. These protocols have been extensively used in epidemiological studies led by IARC in various settings [16].

Dried blood spot (DBS), dried urine strip (DUS), and dried stool cards are an easy and inexpensive means of collection and storage of biospecimens in settings where collection and storage of plasma, urine, and stool are not optimal (e.g., due to poverty, logistic, or environmental/climate constraints). DBS can be used for molecular biology techniques and other diagnostic assays. All of these collection cards can reduce the cost and difficulty of cold chain shipping of samples, and can be shipped as non-dangerous goods at room temperature [17, 18].

Blood samples were collected in TFN and GenSaver cards, stool samples were collected in GenSaver and GenCollect cards, and urine samples were collected in GenCollect cards. 99.5% of the participants provided with both types of blood cards. For GenCollect 82% and for GenSaver 83% of the participants provided stool samples. Urine cards were prepared only for the first hundred participants as a sample. Before sample collection, the following data were recorded from all subjects: date and time of sample collection, date and time of preparing and packing biospecimen collection cards, fasting status (blood samples were only collected during fasting), time since last meal or drink, diet on previous day given that intake of some foods may affect biological measurements, smoking status, last menstrual cycle (women only), and use of any type of drugs or dietary supplements (i.e., multivitamins) in past week. All DBS, DUS, and dried stool samples were stored in a clean, dry, temperature, and humidity-controlled area of the laboratory, not exceeding 30 °C and with no direct sunlight exposure before their shipment to IARC for storage and biomarker analyses.

#### Blood Samples

Blood samples were obtained by venipuncture from fasting participants seated in a semi-upright position into two vacutainers tubes of 5 ml containing heparin anticoagulant. One tube was analysed for lipid and glucose levels and the second tube was used for the preparation of DBS on one GenSaver and two BioSample TFN collecting cards (Ahlstrom). Using a pipette with a disposable tip, 125 μl and 50 μl of blood (for Gensaver and TFN, respectively) was transferred by the laboratory technician to the centre of one circle on a labelled filter card without touching the filter paper directly with the tip of the pipette. This procedure was repeated to fill all circles of the cards and three cards were prepared per participant. The paper cards were then let dry for 4 h at room temperature and later placed in a labelled plastic bag with a desiccant sachet with a humidity indicator. DBS were stored at room temperature before shipment to IARC.

#### Stool Samples

All stool samples were collected at the study recruitment site, using a standard protocol: stool collection paper was placed inside the toilet bowl. The participant collected a sample of the stool with a spatula, placed it in a labelled stool collection cup, and returned the container to the laboratory. There, a small portion of stool was scraped by the laboratory technician using a wooden stick and all four cells of the card were smeared thinly with stool and the flap was closed. For each participant, two stool cards, a GenSaver and a GenCollect, were collected. When completed, the stool cards were inserted into a gas-impermeable labelled plastic bag containing a desiccant pack with a humidity indicator.

#### Urine Samples

Participants provided urine samples in separate clean plastic containers along with stool samples. GenCollect urine card was soaked with urine in the container until it was completely saturated. The card was then suspended in a secure place and allowed to dry for around 6 h. Finally, the dried card was covered with the flap and sealed in a labelled plastic bag with a humidity indicator desiccant pack. Two DUS cards were prepared for each participant. Before their shipment to IARC, these samples were kept in a temperature not exceeding 30 °C and humidity-controlled (no more than 22%) room, safe from direct exposure to sunlight.

#### Biomarker Analyses to Measure Metabolic Health

Glucose and lipid profile (total cholesterol, triglyceride, HDL-cholesterol, and LDL-cholesterol) measurements were performed in plasma from fasting participants in Kandahar University Teaching Hospital. Samples were centrifuged and analysed using an enzymatic photometric test method within 1 h of sample collection. Participants with a fasting plasma glucose level of > 115 mg/dL or on antidiabetic medicine were considered diabetics. Dyslipidaemia was defined as triglycerides > 200 mg/dL, total cholesterol > 200 mg/dL, LDL-C > 130 mg/dL or HDL-C < 40 mg/dL [19, 20].

#### Future Biomarker Analyses

The establishment of a biobank, which includes DBS, DUS, and dried stool samples, will be of great value to perform high-quality biomarker-based research, such as metabolomics and microbiome analyses on stool and blood samples and DNA extraction from DBS for genetics and epigenetics studies.

All questionnaire data and biological samples were centralized at the International Agency for Research on Cancer in Lyon, France.

### Data Handling and Statistical Analysis

#### Data Cleaning

All data of the questionnaire were entered into Epi Info 7. The electronic version of the database was double-checked with paper forms which were completed manually during interviews. A unique numerical identifier was given to each study participant. Data variables were properly coded and labelled for statistical analyses. Duplicate observations and invalid data points were removed and spelling errors were corrected.

#### Statistical Analyses

We will assess the association between demographic risk factors, dietary patterns, and biological markers and metabolic health. All potential confounders available will be adjusted for in each analysis. Logistic regression and standard normal regression will be used to study the association between the exposures of interest and the risk of obesity. Statistical analyses are performed at Kandahar University and at IARC by STATA version 14, SPSS version 23 and SAS 9.4.

## Preliminary Results

Major descriptive characteristics of the study population are presented in Table 2. A total of 711 subjects, 302 (42%) women and 409 (58%) men, were included in the analyses. As we used stratified sampling, approximately the same proportion of normal weight (36.2%), overweight (32.8%), and obese (31.0%) participants were recruited. Obesity was, however, significantly higher in women (44.7%) than in men (21%). Mean age at recruitment was 37.4 years, higher for women than men (42 vs 34). A majority of the study population was 20–30 years of age (42.9%), living in urban area (92.3%), and married (73.1%), whereas half of the participants (50.8%) were not educated. Mean BMI and waist circumference were 29.5 kg/m^2^ and 97.3 cm among women, and were higher than men, 26.1 kg/m^2^, and 93.7 cm, respectively. 8.6% of the participants were tobacco smokers, higher in men (13.2%) than in women (2.3%), while tobacco snuff users were 14.8%, also higher in men (22.3%) than in women (4.6%). Women were more active than men in moderate activity, while vigorous and light physical activities were higher among men.

**Table 2:** Major descriptive characteristics of the study population according to gender

Variables^1^	Gender		Total (*n* = 711)	*P* value^2^
	Female	Male		
	302 (42%)	409 (58%)		
Mean (STD)				
Age (years)	42.0 (13.4)	34.0 (13.2)	37.4 (13.8)	< 0.001
People in the house	13.9 (10.5)	10.4 (8.4)	12 (9)	< 0.001
Weight (kg)	71.2 (15.4)	76.6 (16.1)	74.3 (16.0)	< 0.001
Height (cm)	155.4 (5.4)	171.2 (5.9)	164.5 (9.7)	< 0.001
Sitting height (cm), missing = 1	79.6 (3.7)	89.5 (3.4)	85.3 (6.0)	< 0.001
Waist circumference (cm)	97.3 (14.9)	93.7 (14.5)	95.2 (14.8)	< 0.001
Hip circumference (cm)	106.3 (13.3)	100.5 (9.6)	103.0 (11.7)	< 0.001
Body mass index (kg/m^2^)	29.5 (6.4)	26.1 (5.1)	27.5 (5.9)	< 0.001
Waist-to-hip ratio (WHR)	0.91 (0.07)	0.93 (0.08)	0.92 (0.08)	0.016
Weekly physical activity				
Vigorous activity (METs)	160.5 (514)	337.6 (1209)	262.4 (980)	0.017
Moderate activity (METs)	1586.5 (1577)	376.3 (958)	890.3 (1393)	< 0.001
Light activity (METs)	292.5 (556)	696.1 (841)	525.6 (760)	< 0.001
Total activity (METs)	2039.5 (1780)	1410.0 (1829)	1677.3 (1829)	< 0.001
Daily sitting time (h)	9.6 (3)	11.0 (3)	10.4 (3)	< 0.001
Daily sleeping time (h)	8.2 (1.6)	7.4 (1.3)	7.7 (1.5)	< 0.001
Anxiety/depression score (HSCL-25)	2.25 (0.6)	1.58 (0.4)	1.87 (0.6)	< 0.001
*N* (%)				
BMI classes				< 0.001
Normal weight	78 (25.8)	179 (43.8)	257 (36.2)	
Overweight	89 (29.5)	144 (35.2)	233 (32.8)	
Obese	135 (44.7)	86 (21.0)	221 (31.0)	
Age in categories				< 0.001
20–30	76 (25.1)	229 (56.0)	305 (42.9)	
31–40	69 (22.9)	65 (15.9)	134 (18.9)	
41–50	91 (30.1)	62 (15.2)	153 (21.5)	
50+	66 (21.9)	53 (12.9)	119 (16.7)	
Living address				0.301
Rural	27 (8.9)	28 (6.9)	55 (7.7)	
Urban	275 (91.1)	381 (93.1)	656 (92.3)	
Marital status				< 0.001
Married	241 (79.8)	279 (68.2)	520 (73.1)	
Single	19 (6.3)	129 (31.5)	148 (20.8)	
Widow/separated	42 (13.9)	1 (0.3)	43 (6.1)	
Educational status				< 0.001
None	269 (89.1)	92 (22.5)	361 (50.7)	
Up-to high school	28 (9.3)	126 (30.8)	154 (21.7)	
Higher education	5 (1.7)	191 (46.7)	196 (27.6)	
Occupational categories				< 0.001
Non-manual workers	4 (1.3)	150 (36.7)	154 (21.7)	
Farm workers	0 (0)	20 (4.9)	20 (2.8)	
Manual workers	1 (0.3)	63 (15.4)	64 (9.0)	
Students	3 (1)	148 (36.2)	151 (21.2)	
Housewife/jobless	294 (97.4)	28 (6.8)	322 (45.3)	
Smoking status				< 0.001
Never smoker	276 (92.4)	285 (69.7)	561 (78.9)	
Former smoker	19 (6.3)	70 (17.1)	89 (12.5)	
Current smoker	7 (2.3)	54 (13.2)	61 (8.6)	
Snuffing status				< 0.001
Never	285 (94.4)	306 (74.8)	591 (83.1)	
Former	3 (1.0)	12 (2.9)	15 (2.1)	
Current	14 (4.6)	91 (22.3)	105 (14.8)	
Central obesity				< 0.001
No	46 (15.2)	212 (51.8)	258 (36.3)	
Yes	256 (84.8)	197 (48.2)	453 (63.7)	
Hypertension				< 0.001
No	143 (47.4)	309 (75.6)	452 (63.6)	
Yes	159 (52.6)	100 (24.4)	259 (36.4)	
Diabetes mellitus				0.07
No	243 (80.5)	350 (85.6)	593 (83.4)	
Yes	59 (19.5)	59 (14.4)	118 (16.6)	
Dyslipidaemia				0.001
No	152 (50.3)	256 (62.6)	408 (57.4)	
Yes	150 (49.7)	153 (37.4)	303 (42.6)	
Fatty liver				< 0.001
No	166 (55.0)	288 (70.4)	454 (63.9)	
Yes	136 (45.0)	121 (29.6)	257 (36.1)	
Anxiety/depression (HSCL-25)				< 0.001
No	29 (9.6)	208 (50.9)	237 (33.3)	
Probable	32 (10.6)	88 (21.5)	120 (16.9)	
Case	241 (79.8)	113 (27.6)	354 (49.8)	

Continuous variables are shown with mean and standard deviation; categorical variables are shown with number and percentage

^1^Number of missing values is 0 unless otherwise specified

^2^Chi-square test or ANOVA

Parameters of metabolic health and stress were higher in women than in men. Among women, the prevalence of central obesity, hypertension, diabetes mellitus, and dyslipidaemia was 84.8%, 52.6%, 19.5%, and 49.7%, while in men, it was 48.2%, 24.4%, 14.4%, and 37.4%, respectively. Mean score of Hopkins Symptoms Checklist (HSCL-25) was 1.87 for both sexes. However, the mean score was 2.25 for women and 1.58 for men. Almost half the participants (49.8%) were symptomatic for anxiety/depression and it was significantly higher in women (79.8%) than in men (27.6%).

## Discussion

This is the first population-based study which will focus on dietary patterns, demographic and lifestyle factors, and their association with obesity and metabolic health in Afghanistan through collection of dietary data and biological samples. Such a study will aid the development of dietary and lifestyle guidelines in Afghanistan which will promote better health and educate people to make healthier food choices.

NCDs are becoming one of the major health issues in a country with more than 3 decades of war, instability, and widespread poverty. Overall, NCDs account for 44% of all deaths in the country [21]. Therefore, NCDs and their modifiable risk factors should be tackled effectively. Meanwhile, obesity is associated with chronic diseases, such as stroke, hypertension, cardiovascular disease, type-2 diabetes, and certain forms of cancer. Obesity, especially when it is ignored and becomes chronic, is not only a problem of individuals, but it is a problem which affects the whole population and even generations. Obesity not only increases the risk of metabolic and chronic diseases, but it also impacts quality of life [22]. Considering recent advances in nutrition science, focusing on diet can be helpful for the prevention of chronic diseases [23].

Very few (cross-sectional) studies have been conducted about overweight/obesity, metabolic health, and NCDs in Afghanistan, and most of them were published after 2010. In a series of cross-sectional studies conducted in the five major cities of Afghanistan, the prevalence of overweight and obesity was 34.3%, 16% in Kandahar, 38.1%, 31.2 in Kabul, 31.8%, 15.8 in Herat, 34%, 15.5% in Mazar-e-Sharif, and 32.1%, 27.4% in Jalalabad, respectively [24–28]. The prevalence of diabetes mellitus was 22.4% in Kandahar, 13.3 in Kabul, 9.9% in Herat, 9.2% in Mazar-e-Sharif, and 11.8 in Jalalabad, respectively [24–27, 29]. A recent systematic review and meta-analysis reported that the overall prevalence of diabetes mellitus was around 12% in Afghanistan, being the highest in Kandahar city, with no significant difference between sexes [29].

No published studies were found regarding the traditional lifestyle and dietary patterns of the Afghan population, and therefore, there is a strong need to conduct studies on current dietary patterns and lifestyle in the country. Nevertheless, Afghanistan’s first national food-based dietary guidelines were published in 2016. The guideline uses a tablecloth for graphical representations of the food groups, while the main food group is represented in the centre with the largest plate. For convenience, all foods were divided into 7 major groups: cereals and tubers; pulses, beans, nuts and seeds; dairy; meat, fish and eggs; fruits; vegetables; and fats and oils. The main objectives of these guidelines are to promote healthy food choices and adequate food intake, and to prevent overweight/obesity [30, 31]. Adherence to these guidelines could be assessed in future dietary surveys.

Through the establishment of a cross-sectional study in Kandahar city, one of the largest provinces of Afghanistan, we will examine the role of different factors such as dietary patterns, physical activity, and lifestyle in the development of obesity and metabolic health. The findings will help in designing and implementing effective public health strategies to promote a healthy lifestyle and prevent the epidemic of overweight and obesity, and hence, reduce the burden of non-communicable diseases in the region.

Furthermore, the establishment of a large high-quality cross-sectional study in Afghanistan has great potential and is an important investment for future research hypotheses. The data collected will constitute an invaluable resource for future studies on lifestyle patterns, biomarkers, and the microbiome, and could be used to train future epidemiologists and public health scientists in Afghanistan and other regions/countries with similar living conditions.

## Data Availability

The datasets used and/or analysed during the current study are available from the corresponding author on reasonable request.
